# Genome Sequence of Oenococcus oeni UNQOe19, the First Fully Assembled Genome Sequence of a Patagonian Psychrotrophic Oenological Strain

**DOI:** 10.1128/MRA.00889-18

**Published:** 2018-08-09

**Authors:** Néstor G. Iglesias, Danay Valdés La Hens, Nair T. Olguin, Bárbara M. Bravo-Ferrada, Natalia S. Brizuela, E. Elizabeth Tymczyszyn, Horacio Bibiloni, Adriana C. Caballero, Lucrecia Delfederico, Liliana Semorile

**Affiliations:** a Laboratorio de Ingeniería Genética y Biología Celular y Molecular, Instituto de Microbiología Básica y Aplicada, Departamento de Ciencia y Tecnología, Universidad Nacional de Quilmes, Bernal, Argentina; b Consejo Nacional de Investigaciones Científicas y Técnicas (CONICET), Buenos Aires, Argentina; c Laboratorio de Microbiología Molecular, Instituto de Microbiología Básica y Aplicada, Departamento de Ciencia y Tecnología, Universidad Nacional de Quilmes, Bernal, Argentina; d Comisión de Investigaciones Científicas de la Provincia de Buenos Aires (CIC-BA), La Plata, Argentina; e Bodega Humberto Canale, General Roca, Río Negro, Argentina; f Facultad de Ciencia y Tecnología de los Alimentos, Universidad Nacional del Comahue and PROBIEN–CONICET, Neuquén, Argentina; Louisiana State University

## Abstract

Oenococcus oeni UNQOe19 is a native strain isolated from a Patagonian pinot noir wine undergoing spontaneous malolactic fermentation. Here, we present the 1.83-Mb genome sequence of O. oeni UNQOe19, the first fully assembled genome sequence of a psychrotrophic strain from an Argentinean wine.

## ANNOUNCEMENT

Oenococcus oeni UNQOe19 is a native psychrotrophic strain isolated from a pinot noir wine undergoing spontaneous malolactic fermentation (MLF) at the oldest commercial winery in General Roca, North Patagonia, Argentina. Pinot noir grapes grow very well in the North Patagonian region due to the agroecological conditions there. In a previous work, we showed the prevalence of O. oeni and Lactobacillus plantarum strains in Patagonian wines undergoing spontaneous MLF, which suggests that both species are involved in leading the MLF process (1). In this work, we report the first fully assembled genome sequence of a psychrotrophic O. oeni strain, UNQOe19, which showed a good capacity for implantation in microvinification assays, driving the MLF at low incubation temperatures. This finding suggests its potential to be used as a malolactic starter culture at environmental temperatures below 15°C (2).

Strain UNQOe19 was grown in Leuconostoc oenos medium (MLO) (3) at 28°C for 7 days. To obtain DNA, 1 mg/ml of lysozyme with 1% sodium dodecyl sulfate was used. Proteins were removed with 0.1 µg/ml of proteinase K, followed by phenol-chloroform-isoamyl alcohol (25:24:1) extraction. A total of 16 µg of high-quality genomic DNA was required for library preparation and sequencing. A whole-genome shotgun library was constructed using a 20-kb SMRTbell version 1.0 template prep kit, followed by single-molecule real-time (SMRT) sequencing conducted on an RS II (Pacific Biosciences) sequencer. A total of 103,710 reads (588-fold coverage and a polymerase read *N*_50_ size of 14,765 bp), with an average length of 10,359 bp and an estimated accuracy of 85%, were used as input for *de novo* assembly with the Canu package (4). The Canu output consisted of a single circular contig without gaps; the chromosomal contig was 1,826,824 bp long with a 37.9% G+C content. Prediction and annotation of the coding sequences were conducted with GeneMarkS (5). Genome annotation was done using the NCBI Prokaryotic Genome Annotation Pipeline (6), and gene ontology relationships were estimated using Blast2GO version 5.1.1 (7). The Bacterial Pan-Genome Analysis (BPGA) pipeline (8) was used to compare the presence/absence of genes in strain UNQOe19 with other O. oeni strains. Out of 1,891 predicted genes, 1,721 were identified as protein-coding DNA sequences, 118 were potential pseudogenes, and 52 were RNA coding genes (43 tRNAs, 6 rRNAs, and 3 noncoding RNAs); these findings are comparable to those for other O. oeni strains (9–13).

Compared with the genome annotation of O. oeni strain PSU-1 (14), 160 unique genes in O. oeni UNQOe19 were detected. Among them, 19 genes were related to cellular components, 29 to metabolic processes, 23 to DNA molecular functions, 11 to plasmid and related conjugal transfer proteins, 13 to transmembrane transporter activities, 21 to putative phage-related proteins, and 38 to hypothetical proteins. Interestingly, the remaining six genes were related to homeostasis and oxidoreductase activity stress response, which is similar to earlier results described for other lactic acid bacterial species (15–17).

Further detailed evaluation will help elucidate the molecular basis of O. oeni strain UNQOe19’s potential as a malolactic starter culture and its ability to adapt to the stressful wine conditions, including low environmental temperatures, in the North Patagonian region.

### Data availability.

The whole-genome sequence of O. oeni strain UNQOe19 has been deposited at GenBank under the accession number CP027431.
